# Direct PIP_2_ binding mediates stable oligomer formation of the serotonin transporter

**DOI:** 10.1038/ncomms14089

**Published:** 2017-01-19

**Authors:** Andreas Anderluh, Tina Hofmaier, Enrico Klotzsch, Oliver Kudlacek, Thomas Stockner, Harald H. Sitte, Gerhard J. Schütz

**Affiliations:** 1 Institute of Applied Physics, TU Wien, Wiedner Hauptstrasse 8-10, Vienna 1040, Austria; 2 Center for Physiology and Pharmacology, Institute of Pharmacology, Medical University Vienna, Waehringerstrasse 13A, Vienna 1090, Austria; 3 EMBL Australia Node in Single Molecule Science, School of Medical Sciences, ARC Centre of Excellence in Advanced Molecular Imaging, University of New South Wales, Sydney, New South Wales 2052, Australia

## Abstract

The human serotonin transporter (hSERT) mediates uptake of serotonin from the synaptic cleft and thereby terminates serotonergic signalling. We have previously found by single-molecule microscopy that SERT forms stable higher-order oligomers of differing stoichiometry at the plasma membrane of living cells. Here, we report that SERT oligomer assembly at the endoplasmic reticulum (ER) membrane follows a dynamic equilibration process, characterized by rapid exchange of subunits between different oligomers, and by a concentration dependence of the degree of oligomerization. After trafficking to the plasma membrane, however, the SERT stoichiometry is fixed. Stabilization of the oligomeric SERT complexes is mediated by the direct binding to phosphoinositide phosphatidylinositol-4,5-biphosphate (PIP_2_). The observed spatial decoupling of oligomer formation from the site of oligomer operation provides cells with the ability to define protein quaternary structures independent of protein density at the cell surface.

## Abstract (web summary)

The human serotonin transporter (SERT) mediates the uptake of neurotransmitters to terminate neuronal signalling. Here the authors use single-molecule imaging to get insight into the molecular origin of SERT oligomerization and their pre-set stoichiometry at the plasma membrane.

The human serotonin transporter (SERT) is a 12-pass transmembrane protein targeted to presynaptic nerve terminals and belongs to the neurotransmitter:sodium symporter (NSS) or solute carrier 6 (SLC6) family^1^. These transmembrane proteins mediate the high-affinity uptake of neurotransmitters from the synaptic cleft and are, hence, of pivotal importance for synaptic signal transmission by terminating chemical signal transduction between neurons. SERT is the target for antidepressants like serotonin-selective reuptake inhibitors^2^ as well as for illicitly used drugs such as amphetamines^3^; the latter act by reversing the transport direction of SERT, provoking release of serotonin (5-HT) into the extracellular space^4^.

Biochemical and spectroscopic studies have reported that SERT is present as oligomeric complexes at the plasma membrane^5^,^6^,^7^,^8^,^9^. Likewise, oligomerization of a number of other NSS family members has been described^10^. The oligomeric state, however, does not seem to be crucial for uptake activity: for example, it was found that oligomerization-deficient mutants of the GABA transporter (GAT1) retain unchanged transport activity^11^. Currently, two possible roles of NSS oligomerization are discussed: (i) oligomerization of correctly folded proteins is necessary to pass the quality control for trafficking from the endoplasmic reticulum (ER)^12^, in the case of SERT specifically by allowing the interaction with SEC24C (refs 13, 14). (ii) It has been reported that oligomerization is a prerequisite for the reverse operation of the transporter which affords substrate release^15^.

Using single-molecule fluorescence microscopy, we have previously discovered that SERT forms a broad distribution of assemblies ranging from monomers up to pentamers^7^. The homo-association at the plasma membrane did not depend on SERT surface density and was stable at least over 10 min. We proposed a model based on kinetic trapping of oligomers at the plasma membrane, subsequent to an equilibration which occurred at an unknown subcellular organelle. The site of equilibration and the mechanism behind kinetic trapping, however, remained unclear.

Some arguments pointed our interest to the negatively charged phospholipid phosphatidylinositol-4,5-biphosphate (PIP_2_). PIP_2_ is part of a number of signalling pathways, for example, endo- and exocytosis, cell adhesion, cell motility, phagocytosis or G protein-coupled receptor signalling^16^. It is a minor phospholipid that is mainly found at the cytoplasmic leaflet of the plasma membrane, where it occurs at a surface density of about *σ*_PIP_ ∼20,000–60,000 molecules μm^−2^ (ref. 17). Pronounced differences exist in the subcellular localization of PIP_2_: PIP_2_ comprises about 1% of total lipid at the plasma membrane^17^, whereas only trace amounts of PIP_2_ are present at the ER^18^. PIP_2_ binding to transmembrane proteins is frequently observed, for example, for ion channels, where it regulates channel activity by influencing the open probability^19^. Likewise, we have recently found that the functional activity of SERT and the dopamine transporter (DAT) was influenced by PIP_2_ binding^20^,^21^: upon enzymatic depletion of PIP_2_ or mutation of identified PIP_2_ binding sites, amphetamine-induced substrate efflux was markedly reduced whereas uptake rates were essentially unaffected. Similarly, while the oligomeric configuration does not seem to influence neurotransmitter uptake^11^, amphetamine-induced neurotransmitter release has been shown to rely on the quaternary arrangement^15^.

In the present study, we quantify the degree of SERT oligomerization at different subcellular localizations of Chinese hamster ovary (CHO) cells and analyse the kinetics of protomer turnover. We find evidence that SERT oligomers are pre-formed at the ER following a dynamic equilibrium model. At the plasma membrane, kinetic trapping arrests the oligomers at the pre-set stoichiometry. Our data suggest that the different subcellular concentrations of PIP_2_ mediate the differential SERT oligomerization behaviour, likely by direct physical connection of SERT protomers.

## Results

### SERT oligomerization depends on subcellular localization

First, we addressed if SERT oligomerization differs between the plasma membrane and the ER. Monomeric GFP (mGFP) was inserted at the cytosolic N-terminus of SERT to allow for visualization (mGFP–SERT)^22^. Plasma membrane-localized mGFP–SERT was recorded at the bottom cell membrane via total internal reflection fluorescence (TIRF) microscopy. To retain SERT in the ER, we overexpressed a dominant-negative mutant of Sar1a (Sar1a-T39N)^13^, a small GTPase that regulates the assembly of COPII vesicles for plasma membrane trafficking. Sar1a-T39N is a GDP-restricted mutant which prevents the formation of COPII in a dominant-negative manner, thereby arresting SERT in the ER. ER-retained mGFP–SERT was studied at junctions between ER and the plasma membrane, where single-molecule tracking at high signal-to-noise ratio using TIRF microscopy is feasible^23^,^24^.

**Figure 1:**
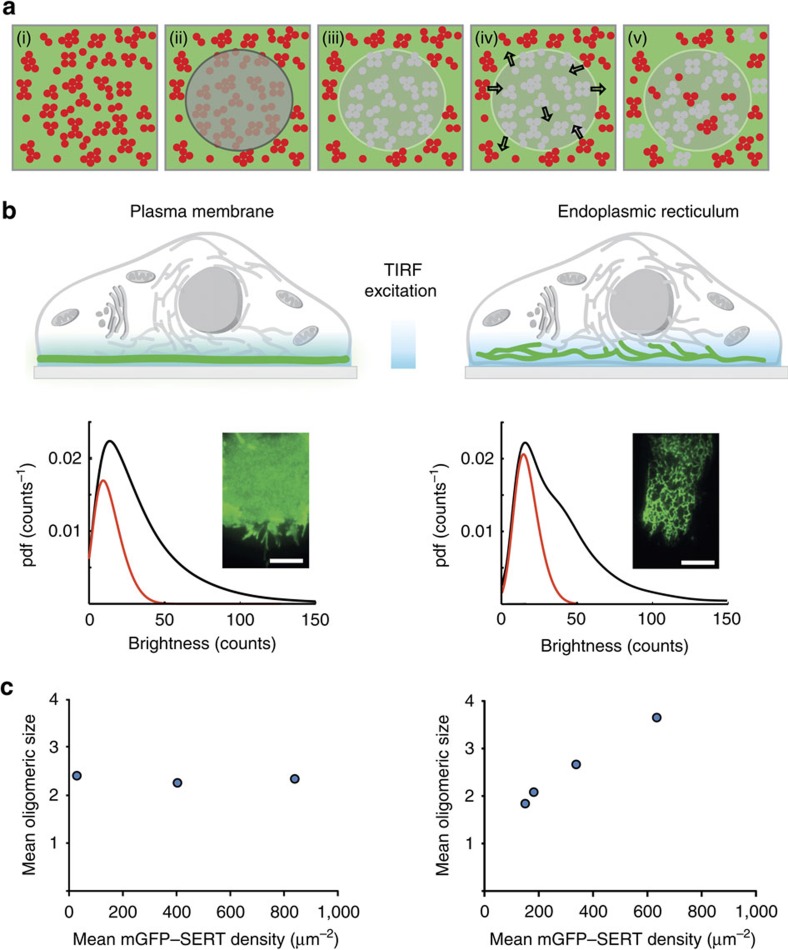
Determination of mGFP-SERT oligomer sizes by single molecule brightness analysis. (**a**) Thinning out clusters while conserving stoichiometry of labeling (TOCCSL). Using a field stop in the laser beam pathway, a small area of the densely fluorescently labelled cell membrane (i) is irreversibly photobleached (ii, iii). During the recovery phase (iv), SERT oligomers diffuse back into the bleached area. At the onset of this process, they can be discriminated as single, well separated fluorescent spots (v). (**b**) The oligomeric state of SERT was evaluated in the plasma membrane (left panel) or the endoplasmic reticulum (right panel) by single-molecule brightness analysis. The brightness distributions (in counts) of mGFP–SERT complexes are plotted as pdf. The plots show the distribution of the complexes from the TOCCSL image after the recovery phase (black curves) and the measured brightness of a monomer (red curves); see also Supplementary Fig. 3. A fit yields the distribution of oligomeric sizes at the respective organelle. Scale bars, 10 μm. (**c**) At the plasma membrane, the mean oligomeric size is independent from the density of SERT (*n*>22 cells per datapoint; plotted protein densities were 29±17 (s.e.m.) μm^−2^, 402±31 μm^−2^ and 840±56 μm^−2^; s.e.m. of the mean oligomeric sizes were smaller than 0.05). In contrast, at the ER higher expression levels correlate with larger oligomeric sizes (*n*>19 cells per datapoint; plotted protein densities are 153±13 (s.e.m.) μm^−2^, 185±24 μm^−2^, 343±37 μm^−2^ and 643±36 μm^−2^; s.e.m. of the mean oligomeric sizes were smaller than 0.05).

We used the ‘Thinning out clusters while conserving stoichiometry' (TOCCSL) technique previously established in our lab^7^,^25^,^26^,^27^ to determine the oligomeric state of mGFP–SERT. TOCCSL extends fluorescence recovery after photobleaching (FRAP) to the level of single-molecule fluorescence microscopy (Fig. 1a). Typically, the high density of fluorescently labelled proteins results in a homogenously labelled surface, thereby precluding direct single-molecule measurements (Fig. 1a,i). In TOCCSL, a small area of the cell membrane is irreversibly photobleached by a strong laser pulse focused through a rectangular pinhole onto the sample (Fig. 1a,ii,iii). The high laser intensity completely abrogates the fluorescence signal of the mGFPs in every SERT molecule within the illuminated area, while retaining full brightness outside this area. During the subsequent recovery phase unbleached molecules and oligomers diffuse into the previously bleached area due to Brownian motion (Fig. 1a,iv). In contrast to FRAP experiments, however, we exploit in TOCCSL the very onset of this recovery process, when individual molecules can be monitored as single, clearly distinguishable fluorescent spots (Fig. 1a,v). The brightness of these spots was determined and compared with the brightness of single mGFP–SERT molecules recorded under the same conditions and in the same subcellular compartments, yielding the statistical distribution of mGFP–SERT oligomers. Note that, similar to fluorescence correlation experiments, TOCCSL allows only analysis of the mobile fraction of molecules.

We used this experimental strategy to determine the mean aggregation size of mGFP–SERT located at the plasma membrane (Fig. 1b, left) or retained at the ER (Fig. 1b, right) of CHO cells. The differences in the subcellular distributions are apparent in TIRF microscopy; while plasma membrane-localized SERT yields a homogenous intensity distribution over the whole interface of the cell with the glass slide (Fig. 1b, left), we observed the characteristic reticular ER–plasma membrane junctions upon Sar1a-T39N overexpression (Fig. 1b, right). The majority of the protein was freely mobile in both cellular compartments, yielding mobile fractions of 82±8% and 66±12% (s.e.m.; *n*≥10 cells) for plasma membrane and for the ER in FRAP experiments, respectively.

We first confirmed our recent finding of SERT oligomerization at the plasma membrane: the left panel of Fig. 1b shows the brightness distribution, plotted as a probability density function (pdf) obtained from the TOCCSL images. A large spread in the oligomer distribution was observed, and the mean oligomeric size did not depend on SERT surface density (Fig. 1c, left). The results were strikingly different, when we determined SERT oligomerization at the ER membrane. While the overall oligomer distribution remained highly heterogeneous (Fig. 1b, right), we found a pronounced increase of SERT oligomer size with increasing mean SERT density at the ER (Fig. 1c, right).

**Figure 2:**
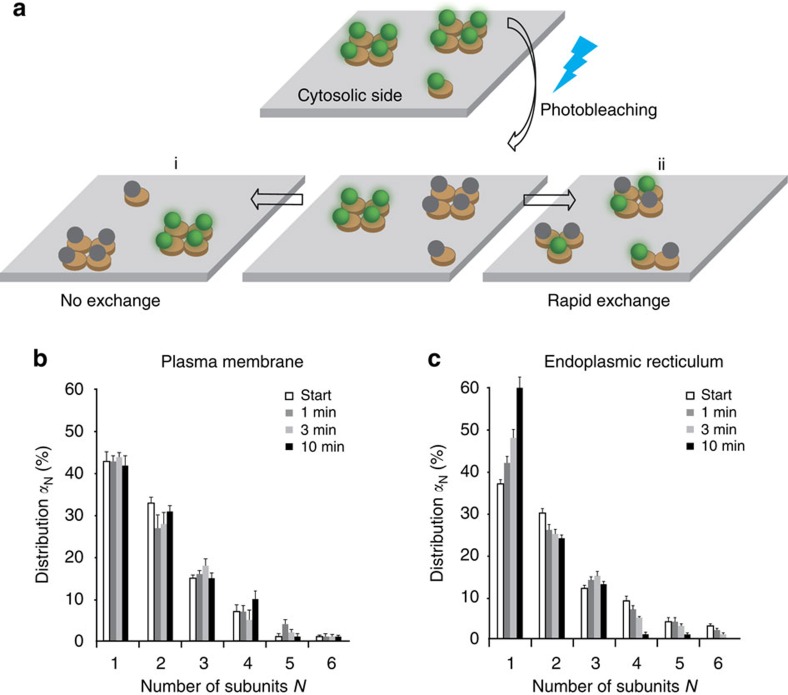
Evaluation of the oligomer stability in the plasma membrane and at the ER. (**a**) To study the stability of SERT oligomers we performed repeated TOCCSL runs on the same cells (1 run per minute over 10 min), and determined the brightness distributions in each run. Two different scenarios can be distinguished: if oligomers were stable over the time course of the experiment, the total number of diffraction-limited spots would be reduced without altering the brightness distribution (left, scenario i). In contrast, if oligomers would exchange subunits during the 10 min, increasing numbers of mixed SERT oligomers containing both bleached and non-bleached subunits would be observable, thereby shifting the determined oligomeric distribution towards smaller structures (right, scenario ii). (**b**) Using the repetitive TOCCSL strategy, we have observed no change in the oligomeric distribution of SERT at the plasma membrane (*n*=20 cells). Oligomeric distributions are shown at the beginning of the experiment (white bars), after 1 min (dark grey), 3 min (light grey), and after 10 min (black). (**c**) At the ER, however, we observed rearrangement of subunits over the timescale of the experiment, as can be seen by the shift of the distributions towards lower oligomer sizes (*n*=22 cells). Error bars show the s.e.m.

A second hallmark of dynamic size equilibration would be the exchange of subunits. To discriminate between stable association and rapid subunit exchange, we used the previously established method of repetitive TOCCSL runs^7^ (Fig. 2a). We performed one run per minute (each consisting of a single bleaching pulse and a single recovery image) over 10 min on the very same cell. Pooling data from multiple cells provided brightness distributions as a function of time. By this procedure, the amount of active fluorophores per cell was substantially reduced to about 50%. In this approach, stable interaction of subunits would reduce the total number of observed spots, but would not alter the brightness distribution (Fig. 2a, scenario i). If the exchange rate of subunits was high, however, bleached subunits would mix with unbleached subunits. Over time, this mixing would increase the number of complexes containing both dark and fluorescent subunits, thereby shifting the observable oligomeric distribution towards smaller structures (Fig. 2a, scenario ii). At the plasma membrane, we observed no change in the oligomeric state with increasing number of TOCCSL runs. We thereby confirmed our previous results which indicated stability of SERT oligomers at the minutes timescale (Fig. 2b). At the ER membrane, however, we found a substantial shift towards oligomers with smaller amounts of active fluorophores, indicating rapid exchange of subunits between SERT oligomers (Fig. 2c).

### Availability of PIP_2_ affects SERT oligomerization

**Figure 3:**
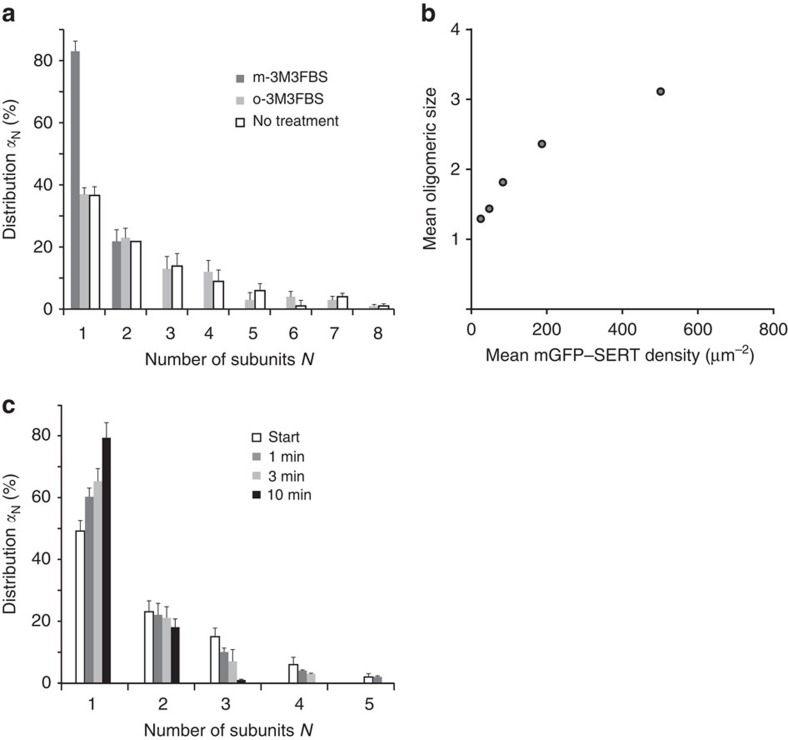
SERT oligomerization at the plasma membrane depends on PIP_2_ levels. (**a**) We enzymatically depleted PIP_2_ at the plasma membrane via activation of phospholipase Cγ (PLCγ) by incubating cells for 15 min with the direct PLCγ-activator *m*-3M3FBS (10 μM). This led to a marked shift of SERT complex sizes towards monomers (dark grey bars). As a negative control, incubation with the inert orthologue *o*-3M3FBS did not yield any effect (light grey bars) in comparison to the untreated cells (white bars) (*n*>20 cells per experimental condition). SERT surface densities were similar: 25±14 (s.e.m.) μm^−2^ (dark grey bars), 38±22 μm^−2^ (light grey bars), 29±17 μm^−2^ (white bars). (**b**) PIP_2_ depletion via m-3M3FBS resulted in marked dependence of mGFP–SERT oligomerization on mGFP–SERT surface density (*n*>20 cells per datapoint; plotted protein densities are 25±14 (s.e.m.) μm^−2^, 48±24 μm^−2^, 84±17 μm^−2^, 187±28 μm^−2^ and 501±40 μm^−2^; s.e.m. of mean oligomeric sizes were smaller than 0.05). (**c**) To test for the effect of PIP_2_ depletion on the stability of the oligomers at the plasma membrane, we performed repetitive TOCCSL runs upon incubating cells with m-3M3FBS (1 μM) (*n*=19 cells). Now, the SERT complexes showed rapid subunit rearrangement, indicating liberation of SERT oligomers from kinetic trapping. The SERT surface density was 89±21 (s.e.m.) molecules μm^−2^. Error bars show the s.e.m.

Given the described SERT–PIP_2_ interaction^20^ and the strong difference of PIP_2_ levels between plasma membrane^17^ and ER^18^, we hypothesized that PIP_2_ impacts on SERT oligomerization. We measured SERT oligomerization at the plasma membrane after depleting PIP_2_ levels via activation of phospholipase Cγ (PLCγ) using the direct PLCγ-activator *m*-3M3FBS (ref. 28). PIP_2_ depletion had no influence on the mean mGFP–SERT surface density (Supplementary Fig. 1). For low SERT surface densities (25 molecules μm^−2^), we observed a marked shift of SERT oligomers towards smaller complexes, whereas application of the inert orthologue *o*-3M3FBS did not elicit any effect (38 molecules μm^−2^, Fig. 3a). At higher SERT densities, however, oligomers increased in size (Fig. 3b), indicating rapid equilibration of the oligomerization reaction.

A consequence of rapid equilibration would be the continuous exchange of subunits between SERT oligomers. Hence, to test for the stability of oligomers we performed repetitive TOCCSL runs after incubating cells with *m*-3M3FBS. The quaternary arrangement of SERT in oligomers now showed rapid subunit exchange (Fig. 3c), which indicated that SERT oligomers were indeed liberated from kinetic trapping. Together, these results show that PIP_2_ depletion results in equilibration of SERT oligomerization and concomitant subunit exchange at the plasma membrane. Of note, SERT oligomerization at the plasma membrane lacking PIP_2_ resembles the oligomerization behaviour at the ER membrane (Fig. 2).

### PIP_2_ binds directly to SERT and mediates oligomerization

**Figure 4:**
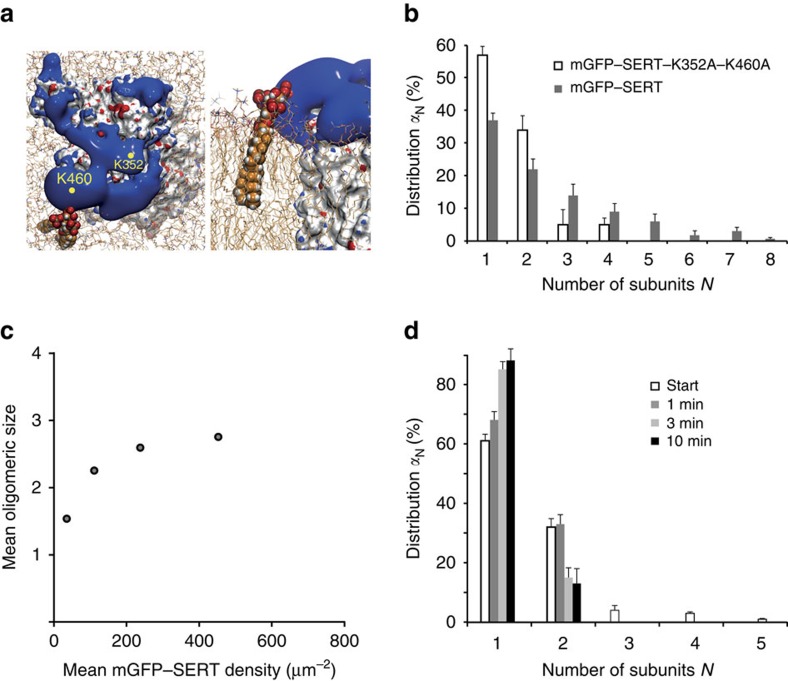
Direct binding of PIP_2_ to SERT mediates oligomerization. (**a**) Analysis of the electrostatic field generated by SERT. The final structure of a 100 ns simulation of a membrane-inserted SERT is shown as viewed from the cytosole (left) or in side-view (right). SERT surface is shown in white, the electrostatic isosurfaces in red (negative potential) and blue (positive potential). For illustration purposes, a PIP_2_ molecule was placed into the membrane (in space filled representation) in close proximity to the large positively charged area that includes residue K460. (**b**) We determined the quaternary assembly of the mutant mGFP–SERT–K352A–K460A at the plasma membrane (white bars). Proteins were expressed at a surface density of 83±31 (s.e.m.) molecules μm^−2^. A distinctive shift to monomers and dimers compared with the wild type (grey bars) was observed (*n*=23 cells). (**c**) A pronounced dependence of mean oligomeric state on mGFP–SERT–K352A–K460A surface density was observed, which saturates around 2.8 transporter molecules per oligomer (*n*>19 cells per datapoint; plotted protein densities are 35±11 (s.e.m.) μm^−2^, 110±33 μm^−2^, 237±14 μm^−2^ and 452±35 μm^−2^; s.e.m. of mean oligomeric sizes were smaller than 0.05). (**d**) Repetitive TOCCSL runs revealed rapid protomer exchange kinetics for this mutant (*n*=18 cells). Error bars show the s.e.m.

Next, we investigated whether direct PIP_2_ binding to SERT sustained oligomer formation or whether PIP_2_ would exert an indirect effect due to the downstream metabolites IP_3_ or DAG. Recently, we identified the amino acids K352 and K460 as crucial residues for PIP_2_ binding to SERT^20^ (Fig. 4a); mutation of both residues to alanine yielded a substantial decrease of PIP_2_-induced effects on parachloroamphetamine-induced, SERT-mediated current and efflux. Most importantly, the PIP_2_–SERT interaction was shown to be greatly reduced in pull-down experiments^20^. We used an mGFP-fusion construct of this mutant to evaluate the effect of reduced PIP_2_ binding on the oligomeric state. The mGFP-tagged SERT–K352A–K460A double mutant was efficiently trafficked to the plasma membrane and showed similar uptake activity as the wild type^20^. Single-molecule brightness analysis yielded an oligomeric distribution that differed from wild-type SERT (Fig. 4b): the dominant species are monomers and dimers, while the fraction of trimers and tetramers was reduced to almost baseline levels. The double mutant showed a pronounced density dependence of its oligomeric assembly, which seemed to saturate at a level of ∼2.8 transporter molecules per oligomer (Fig. 4c). Repetitive TOCCSL runs revealed rapid protomer exchange (Fig. 4d). Together, mGFP–SERT K352A–K460A behaved similar as wild-type SERT after PIP_2_ depletion.

## Discussion

Although there is a wealth of data supporting the existence of neurotransmitter transporters in oligomeric quaternary structures^10^, the nature of the interaction between the subunits has yet not been unravelled. Here, we examined the size and stability of oligomeric complexes of SERT at two different subcellular localizations, the ER membrane and the plasma membrane. We found that dynamic equilibration of SERT oligomers occurs at the ER membrane. After trafficking through the secretory pathway, the pre-formed oligomers undergo kinetic trapping at the plasma membrane. Pre-equilibration of subunit binding at the ER membrane and kinetic trapping of oligomerized protomers at the plasma membrane enables the cell to spatially decouple the oligomerization process from the final site of oligomer operation. This appears crucial to render the degree of oligomerization insusceptible to different SERT concentrations at various localizations on the plasma membrane.

Our data indicate that the phosphoinositide PIP_2_ plays an essential role in this process and that the different PIP_2_ concentrations of the ER membrane and the plasma membrane are responsible for the pronounced differences. While other phosphoinositides would also be plausible candidates for mediating charge-induced SERT oligomerization, some lines of evidence indicate the specific role of PIP_2_ in this process. First, PLCγ is a highly specific enzyme for PIP_2_ hydrolysis, leaving other phosphoinositides virtually unaffected^29^. Experiments shown in Fig. 3 hence reveal the specific contribution of PIP_2_ to SERT oligomerization. Second, PIP_2_ is by far the most common phosphoinositide in the plasma membrane^16^, thereby outcompeting other phosphoinositide species by shear concentration effects. Third, due to rapid dephosphorylation the ER membrane contains phosphatidylinositol (PI) and phosphatidylinositol(4)phosphate (PI(4)P), but virtually no PIP_2_ or other multi-phosphorylated phosphoinositides^16^. Figures 1 and 2 thus render a role of PI and PI(4)P in SERT oligomerization unlikely.

**Figure 5:**
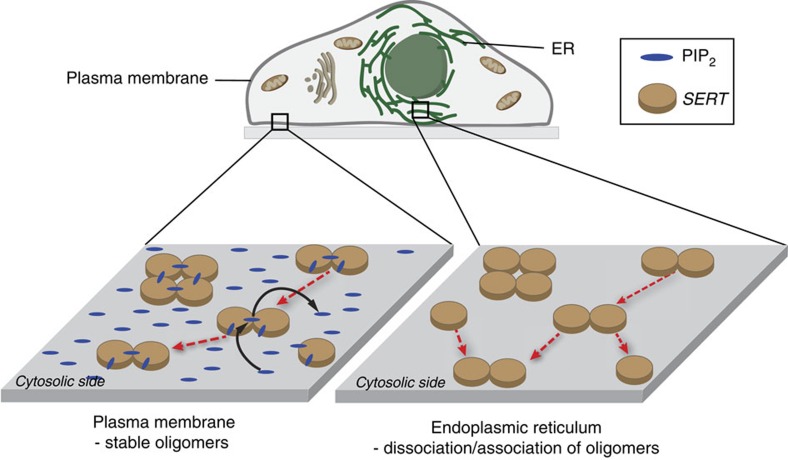
A model for PIP_2_ dependent oligomerization of SERT. High PIP_2_ concentrations at the plasma membrane (left) saturates PIP_2_ binding sites on SERT, impeding further oligomerization of the subunits. Also disassembly of the oligomers is efficiently prevented: in case of PIP_2_ unbinding, the vacant position is rapidly re-populated by a new PIP_2_ molecule before the protomers can separate by diffusion. Together, the two effects lead to the kinetic trapping of the oligomeric state at the plasma membrane. At the ER membrane, however, low PIP_2_ concentrations lead to coexistence of PIP_2_-ligated and -unligated SERT (right), which are capable of mutual binding. Hence, such conditions enable fast equilibration of the oligomerization process, including subunit exchange between SERT oligomers.

In the following, we propose a model how PIP_2_ may cause kinetic trapping of SERT oligomers at the plasma membrane (Fig. 5), which has three cornerstones:

- two negatively charged phosphate groups on PIP_2_ bind electrostatically to positively charged patches on the cytosolic face of two SERT moleculesSERT contains a patch with a strong positive electrostatic potential on the intracellular face which is in contact with the polar headgroups of the membrane^20^ (Fig. 4b). The cationic patch is generated by basic amino acid residues (including K352 and K460). The inositol sugar ring of PIP_2_ has two functional PO_4_^−2^ groups in position 4 and 5, each harbouring a negative charge of −2 (ref. 30). These functional PO_4_^−2^ groups are oriented towards opposing ends of the inositol ring and would therefore have the possibility to interact with two separate SERT monomers. Thereby, they would effectively act as an ionic bridge between the two SERT transporters.
- abrogation of PIP_2_ binding leads to rapid equilibration of the oligomerization process The oligomerization process of SERT rapidly equilibrates under conditions, where PIP_2_ does not contribute to the subunit association process. This was inferred from the results of three different approaches: the depletion of PIP_2_ by enzymatic conversion to IP_3_ and diacylglycerol by activation of PLCγ (Fig. 3); the retention of SERT in the ER, where PIP_2_ levels are extremely low^31^ (Figs 1c and 2c); the reduction of PIP_2_ binding by introduction of two point mutations^20^ (Fig. 4). All conditions revealed qualitatively similar behaviour, that is, the mean size of SERT oligomers became dependent on SERT surface density, and protomers exchanged rapidly between different oligomers. Intrinsic low affinity protein–protein interactions between SERT protomers seem to mediate this process, however, contributions of low PIP_2_ concentrations cannot be ruled out. Of note, equilibration of receptor oligomerization at the ER membrane was recently reported for a GPCR^32^.At this stage, we do not know at which exact subcellular location oligomerization is ultimately adjusted; low PIP_2_ levels in virtually all subcellular membranes except for the plasma membrane suggest that the exchange of subunits remains rapid until the fusion of the cargo vesicles with the plasma membrane.
- elevated PIP_2_ levels saturate PIP_2_ binding sites on SERT, thereby stabilizing the oligomer

In principle, PIP_2_ binding to the polybasic patch at the cytosolic face of SERT, which includes the lysine residues in positions 352 and 460, increases the affinity to other, undecorated SERT protomers. Naturally occurring concentrations of PIP_2_ at the plasma membrane (∼20,000–60,000 molecules μm^−2^; (ref. 33)), however, lead to saturation of SERT with PIP_2_, thereby imposing a charge-based repulsive interaction which precludes further oligomerization.

In consequence, over time most oligomers would disassemble to monomeric SERT until equilibrium is reached, where virtually only PIP_2_-decorated monomers would be present. Importantly, however, there are mechanisms which strongly slow down this equilibration process (‘kinetic trapping'). In fact, the high PIP_2_ concentration itself may account for such a mechanism. Following this line of argumentation, SERT disassembly may require the complete dissociation of one or several PIP_2_ molecules. The high PIP_2_ concentrations at the plasma membrane would result in immediate replenishment of the vacant position before separation of the individual protomers.

Hence, according to our model PIP_2_ levels are not responsible for tuning the oligomeric distribution of SERT, but instead determine the kinetics for reaching the equilibrium of the oligomerization process: at low PIP_2_ levels (as present at the ER membrane) equilibration is fast, whereas at high PIP_2_ levels (as present at the plasma membrane) equilibration is substantially slowed down.

In summary, our data show two important steps in the oligomerization of a transmembrane protein: (i) pre-equilibration of subunit binding at the ER membrane and (ii) kinetic trapping of oligomerization at the plasma membrane. By this, the oligomerization process becomes spatially decoupled from the final site of oligomer operation. This could be important to make the degree of oligomerization insusceptible to different SERT concentrations at various locations on the plasma membrane.

## Methods

### mGFP–SERT construct

eGFP in the peGFP-C1 vector (Clontech) was converted to mGFP^34^,^35^ by mutating alanine 207 of eGFP to lysine using Quik-Change mutagenesis Kit XL from Agilent technologies (Primer: Fw: 5′-TAC CTG AGC ACC CAG TCC AAA CTG AGC AAA GAC CCC AAC-3′, rev: 5′-GTT GGG GTC TTT GCT CAG TTT GGA CTG GGT GCT CAG GTA-3′). cDNAs of WT human SERT and the mutated version SERT-K352A–K460A (ref. 20) were cloned to the mutated vector via HindIII and XhoI restriction sites. For full sequence see Supplementary Note 1. Functionality of mGFP–SERT and mGFP–SERT–K352A–K460A was proven by uptake experiments as described before^20^,^36^.

### Cell culture

CHO cells were cultured at 37 °C and 5% CO_2_ in Dulbecco's modified Eagle's medium (DMEM, PAA Laboratories) supplemented with 10% FCS (Invitrogen), penicillin and streptomycin. Cell lines are tested monthly for mycoplasma contamination using MycoAlert (Lonza).

### Generation of stable cell lines

A CHO cell line (European Collection of Authenticated Cell Cultures) stably expressing mGFP–SERT was generated; transient transfection using the FuGENE 6 transfection kit (Promega) was performed according to the manufacturer's instructions. A total of 6 μg DNA per 10 cm dish was used. The cells were cultured at 37 °C and 5% CO_2_ in DMEM F-12 Ham's (DMEM F-12 HAMS, PAA Laboratories) supplemented with 10% FCS (Invitrogen), penicillin and streptomycin. After 5 days, 800 μg ml^−1^ of G418 (Sigma-Aldrich) was added as a selection marker for stably transfected cells. After 4 weeks the surviving cells were FACS sorted according to their emission in the 488 nm laser line. Only cells showing clear mGFP expression were used for further incubation. Polyclonal cultures were used to ensure a sufficient range of expression levels for density dependent experiments. The cells were further cultured in DMEM F-12 HAMS supplemented with 10% FBS, penicillin, streptomycin and 400 μg ml^−1^ of G418.

### Prevention of mGFP–SERT trafficking from the ER

To retain mGFP–SERT in the ER, a dominant-negative mutant of Sar1a (Sar1a-T39N) was used for co-transfection of CHO cells with mGFP–SERT^13^. A quantity of 990 ng of the expression vector encoding Sar1a-T39N was mixed with 10 ng of mGFP–SERT containing plasmid, ensuring that all transfected cells showed retention of mGFP–SERT in the ER. For all experiments, imaging was performed 12–20 h after the transfection, thereby ensuring similar GFP maturation times.

### Coating of glass slides

Proper attachment of the cell lines was ensured by coating the glass slides with fibronectin (Invitrogen) as follows: the slides were cleaned in 70% ethanol supplemented with 2% hydrochloric acid for 15 min and washed three times for 5 min in dH_2_O. 90 μl fibronectin (50 μg ml^−1^ in 1 × PBS) was uniformly distributed on the glass and dried at 50 °C. Unbound fibronectin was removed by washing the glass slides three times with 1 × PBS (PAA Laboratories) before use.

PIP_2_ depletion experiments were performed on glass slides coated with poly-D-lysine (PDL, Sigma Aldrich). Cleaned slides were incubated with 0.1 mg ml^−1^ PDL for 1 h at 37 °C and washed three times before use.

### Microscopy

A 488 nm laser (SAPPHIRE HP, Coherent Inc.) was mode-cleaned using a pinhole and the illumination intensity and timing were adjusted with an acousto-optical modulator (model 1205, Isomet) using a custom written software (Labview, National Instruments). The laser beam was focused onto the back-focal plane of a TIRF objective (NA 1.46, × 100 *α* Plan APOCHROMAT, Zeiss) mounted on an inverted Zeiss Axiovert 200 microscope. The emission light was filtered using appropriate filter sets for GFP and imaged with a back-illuminated liquid nitrogen cooled CCD camera (Micro Max 1300-PB, Roper Scientific). To restrict the excitation and photobleaching area an adjustable slit aperture (Zeiss) was used as field stop.

All experiments were performed at room temperature. Imaging during all experiments was performed using an objective-type TIRF excitation with an excitation power of ∼0.5–0.8 kW cm^−2^ (determined in epi-configuration) and stroboscopic illumination with excitation times of 3 ms.

### Fluorescence recovery after photobleaching

To determine the mobile fraction of mGFP–SERT, an ∼7 × 7 μm area of the bottom plasma membrane or the plasma membrane–proximal ER was irreversibly photobleached, and the fluorescence recovery over time was monitored (*n*>10 cells). Photobleaching and readout were performed in TIRF configuration. Data were analysed using in-house algorithms implemented in Matlab. The central part of the bleached region was evaluated by integrating all counts and normalizing to the pre-bleach image. For calculation of the mobile fraction *α* of mGFP–SERT the resulting curve was fitted with 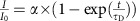.

### TOCCSL

TOCCSL experiments were performed as follows (Supplementary Fig. 2). A pre-bleach image was recorded, which was used for determination of the SERT surface density. After a *t*_pre_=50 ms, a confined region of the cell membrane was photobleached for *t*_bleach_=600–800 ms with a high laser power of ∼ 5–7 kW cm^−2^. To check for complete bleaching, a post-bleach image was recorded *t*_post_=40 ms after the bleach pulse. Finally, the TOCCSL image was recorded after an adjustable recovery time of *t*_recovery_=1,500–12,000 ms. Images were acquired at low excitation power of ∼0.5–0.8 kW cm^−2^ (all excitation intensities were determined in epiconfiguration).

### Brightness analysis

For single-molecule analysis, images were analysed using in-house algorithms implemented in MATLAB (MathWorks). Individual diffraction-limited signals were selected and fitted with a Gaussian intensity profile. The fitting routine yielded the single spot brightness B, which was used to determine the oligomeric state of SERT^7^,^26^,^27^,^37^. The obtained brightness values of each diffraction-limited spot in the TOCCSL image were plotted as a pdf *ρ*(*B*). To obtain the brightness distribution of single mGFP molecules *ρ*_1_(*B*), cells were extensively photobleached, which reduced the amount of active fluorophores to a few molecules per μm^2^, so that each potential mGFP–SERT oligomer contained only one active fluorophore at maximum. By autoconvolution, the monomer brightness distribution was used to calculate the brightness distributions for dimers *ρ*_2_(*B*), trimers *ρ*_3_(*B*) and so on. The overall single spot brightness distribution *ρ*(*B*) was then fitted by a linear combination of *ρ*_1_(*B*), *ρ*_2_(*B*), *ρ*_3_(*B*) and so on:


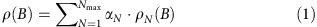


Fitting *ρ*(*B*) with equation (1) yielded the fractions *α*_*N*_ of the different numbers of co-diffusing active mGFP molecules (with the number of mGFP molecules; Supplementary Fig. 3). Note that *α*_*N*_ is proportional to the oligomeric size of SERT, but—due to incomplete mGFP maturation—it slightly underestimates the degree of SERT oligomerization^38^.

At least 750 datapoints were used for *n*-mer calculations. Using simulation approaches, this sample size was shown to be sufficient for obtaining statistically significant results^39^. To determine error bars, we performed a bootstrapping analysis. Briefly, randomly chosen subsamples containing 50% of the data were analysed using equation (1); shown error bars represent the obtained s.d. from 100 repetitions for each oligomeric size divided by 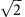.

Mean oligomeric sizes were determined by 
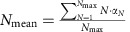
.

### Repetitive TOCCSL experiments

To study oligomer stability, a repetitive TOCCSL protocol was applied. One TOCCSL run per minute was performed on the same region of each cell, following the timing protocol shown in Supplementary Fig. 2. Single runs were repeated over 10 min starting from the first bleach pulse. Both bleaching and image acquisition were done in TIRF mode. The TOCCSL image of each run was used for brightness analysis.

### Determination of the SERT surface density

The mean fluorescence intensity per μm^2^ of the bottom plasma membrane was calculated and divided by the mean single-molecule brightness of mGFP–SERT. To calculate SERT surface densities at plasma membrane–proximal ER, the area fraction of ER–PM junctions was determined from super-resolution images^23^, and the mean intensity of ER-retained SERT was divided by the single-molecule brightness and the respective area fraction.

### Enzymatic PIP_2_ depletion

For activation of phospholipase Cγ, cells were incubated for 20 min at 37 °C with 25 μM 2,4,6-trimethyl-N-[3-(trifluoromethyl)phenyl]benzenesulfonamide (*m*-3M3FBS, Sigma-Aldrich) in Hank's Balanced Salt Solution (HBSS), with calcium and magnesium (Sigma-Aldrich) supplemented with 2% FCS. *m*-3M3FBS remained in the imaging buffer during measurements. For the evaluation of the interaction kinetics (repetitive TOCCSL experiments) a concentration of 1 μM *m*-3M3FBS was used. As a negative control, the inactive ortho-analog *o*-3M3FBS (Tocris) was used.

### Molecular modelling and simulations

The crystal structure of human SERT (PDB ID: 5I6X)^40^ was used as starting structure. The missing side chains were modelled with MODELLER version 9.15 (ref. 41), creating 100 models using the automodel procedure. The best three models, selected according to the DOPE score^42^, were inserted into a 1-palmitoyl-2-oleoyl-sn-glycero-3-phosphatidylcholine containing membrane using the membed procedure^43^. The membrane was pre-equilibrated to contain the SERT transporter^44^. The system was electroneutralized and 150 mM NaCl were added. The environment of SERT was equilibrated while position restraining the transporter. SERT was than released by reducing the position restraints on SERT in four steps, applying 1,000, 100, 10 and 1 kJ mol^−1^, respectively, each time simulating for 2.5 ns. Production runs of 100 ns long equilibrium molecular dynamics simulations were carried out with the GROMACS 5.1 MD package^45^ using the AMBER force field^46^ for the protein and the Berger parameters^47^ for the membrane. The system was maintained at 310 K while coupling protein, membrane and solvent independently using the v-rescale thermostat^48^. The pressure was maintained at 1 bar using the weak coupling algorithm, electrostatic interactions were calculated using the smooth particle mesh Ewald method^49^ with a 1.0 nm cutoff. Lennard–Jones interactions were evaluated applying a 1.0 nm cutoff. Long range corrections for energy and pressure were applied.

### Code availability

The Matlab Source code for TOCCSL analysis is available at https://github.com/schuetzgroup/TOCCSL_analysis.

### Data availability

Data supporting the findings of this study are available within the article and its Supplementary Information Files and from the corresponding author upon reasonable request. The PDB accession code 5I6X (SERT Structure) was used in this work.

## Additional information

**How to cite this article:** Anderluh, A. *et al*. Direct PIP_2_ binding mediates stable oligomer formation of the serotonin transporter. *Nat. Commun.* **8,** 14089 doi: 10.1038/ncomms14089 (2017).

**Publisher's note:** Springer Nature remains neutral with regard to jurisdictional claims in published maps and institutional affiliations.

## Supplementary Material

Supplementary Information Supplementary Figures, Supplementary Note.
