# Supplementary material for: Direct PIP2 binding mediates stable oligomer formation of the serotonin transporter
Source: Nat Commun. 2017 Jan 19;8:14089. doi: 10.1038/ncomms14089 (PMC5253637; doi:10.1038/ncomms14089)
Supplement: Supplementary Information — Supplementary Figures, Supplementary Note. [file ncomms14089-s1.pdf]

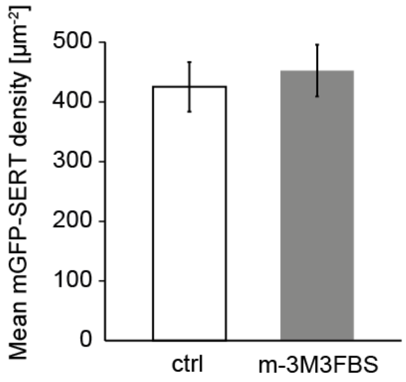

Supplementary Figure 1. Enzymatic depletion of PIP_2 has no influence on the mGFP-SERT surface density. Cells were incubated with 25µM m-3M3FBS for 20min at 37°C. The treatment yields no difference in mGFP-SERT surface density in comparison to untreated cells (n=20). Error bars show the standard errors of the mean (s.e.m.) as evaluated by bootstrapping.

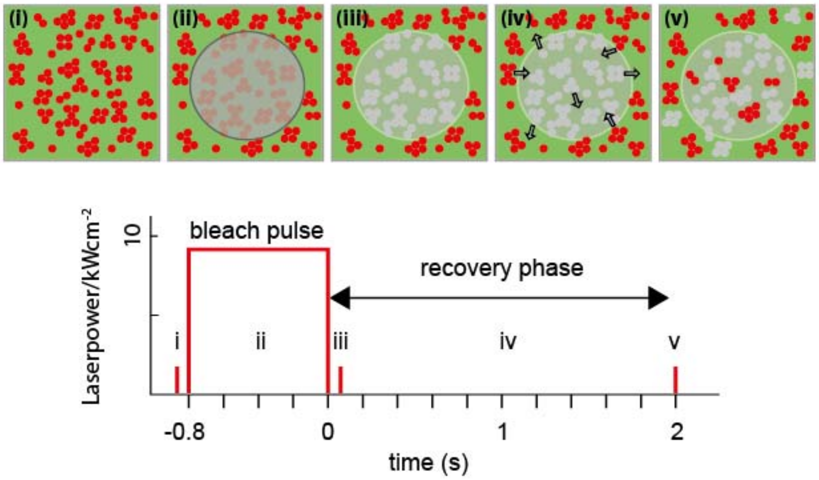

Supplementary Figure 2. The principle of TOCCSL. The upper panel shows a sketch of the plasma membrane during a TOCCSL run, the lower panel shows the corresponding laser protocol. (**i**) First, a pre-bleach image of a cell is recorded at a laser power of ~0.5-0.8 kW/cm^2. This pre-bleach image is used to determine the surface density of mGFP-SERT. (**ii**) A field stop is imaged onto the sample to confine the illumination (grey circle). Using a laser bleach pulse with high intensity (~5-7 kW/cm$^{2)}$, all mGFP molecules within the observed area are irreversibly photobleached. (**iii**) To check for the efficiency of photobleaching an image is recorded 40 ms after the bleach pulse. (**iv**) During a recovery phase of ~ 2s fluorescent spots enter the bleached area due to Brownian motion. (**v**) In the TOCCSL image recorded after the recovery phase, mGFP-SERT complexes can be observed as diffraction-limited signals.

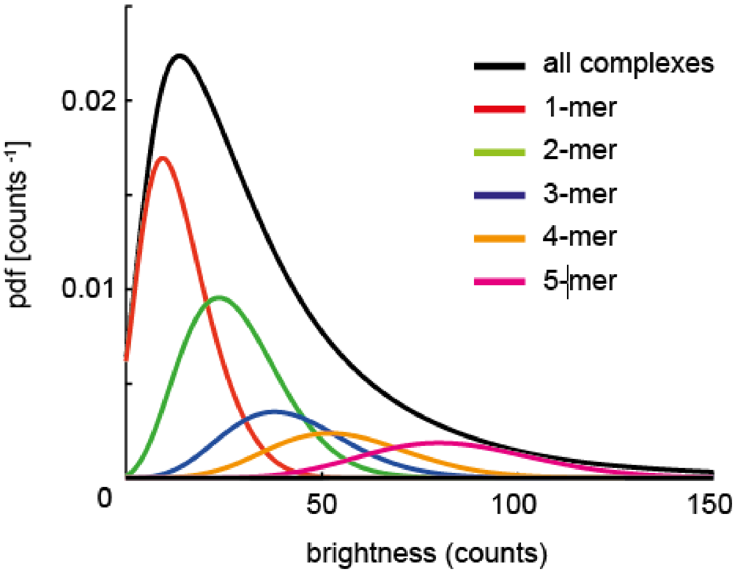

Supplementary Figure 3. Brightness analysis of diffraction limited spots. The obtained brightness distribution of the oligomeric fractions recorded in the TOCCSL image were plotted as probability density function (black line). The red line shows the measured brightness distribution of a monomer. The brightness distributions of dimers (green), trimers (blue), tetramers (orange) and pentamers (pink) were calculated based on the monomer signals by a series of convolution integrals as described in the methods section. The recorded oligomeric data were fitted to Eq. 1, yielding the weighted contribution of the individual n-mers.

MVSKGEELFTGVVPILVELDGDVNGHKFSVSGEGEGDATYGKLTLKFICTTGKLPVPWPTLVTTLTY
GVQCFSRYPDHMKQHDFFKSAMPEGYVQERTIFFKDDGNYKTRAEVKFEGDTLVNRIELKGIDFKED
GNILGHKLEYNYNSHNVYIMADKQKNGIKVNFKIRHNIEDGSVQLADHYQQNTPIGDGPVLLPDNHY
LSTQS**K**LSKDPNEKRDHMVLLEFVTAAGITLGMDELYKSGLRSRAMETTPLNSQKQLSACEDGEDC
QENGVLQKVVPTPGDKVESGQISNGYSAVPSPGAGDDTRHSIPATTTTLVAELHQGERETWGKKVDF
LLSVIGYAVDLGNVWRFPYICYQNGGGAFLLPYTIMAIFGGIPLFYMELALGQYHRNGCISIWRKICPI
FKGIGYAICIIAFYIASYYNTIMAWALYYLISSFTDQLPWTSCKNSWNTGNCTNYFSEDNITWTLHSTS
PAEEFYTRHVLQIHRSKGLQDLGGISWQLALCIMLIFTVIYFSIWKGVKTSGKVVWVTATFPYIILSVL
LVRGATLPGAWRGVLFYLKPNWQKLLETGVWIDAAAQIFFSLGPGFGVLLAFASYNKFNNNCYQDA
LVTSVVNCMTSFVSGFVIFTVLGYMAEMRNEDVSEVAKDAGPSLLFITYAEAIANMPASTFFAIIFFL
MLITLGLDSTFAGLEGVITAVLDEFPHVWAKRRERFVLAVVITCFFGSLVTLTFGGAYVVKLLEEYAT
GPAVLTVALIEAVAVSWFYGITQFCRDVKEMLGFSPGWFWRICWVAISPLFLLFIICSFLMSPPQLRLF
QYNYPYWSIILGYCIGTSSFICIPTYIAYRLIITPGTFKERIIKSITPETPTEIPCGDIRLNAV

Supplementary Note 1. Full amino acid sequence of the mGFP-SERT construct. The N-terminal mGFP sequence is marked in green, the A207K exchange is marked in yellow, the linker sequence is marked in red, and the SERT sequence is shown in white. The two lysines mutated in the PIP_2 binding-deficient mutant (K352A-K460A-mGFP-SERT) are marked in blue.
